# Efficacy and safety of collagenase *Clostridium histolyticum* in men who have sex with men

**DOI:** 10.1093/sexmed/qfag028

**Published:** 2026-05-04

**Authors:** Jake A Miller, Muhammed A Moukhtar Hammad, Lucas G C R de Amorim, Laura Angulo-Llanos, Helen L Bernie, Jeremiah R Dallmer, Grace M Garrett, Gal S Grunhaus, Beatriz S Hernandez, Tung-Chin Hsieh, Bruce R Kava, Mohit Khera, Thomas A Masterson, Vi Nguyen, Thairo A Pereira, Daniela O Rendon, Max D Sandler, Dan V Tran, Faysal A Yafi

**Affiliations:** Department of Urology, University of California, Irvine, Orange, CA 92868, United States; Department of Urology, University of California, Irvine, Orange, CA 92868, United States; Department of Urology, Indiana University School of Medicine, Indianapolis, IN 46202, United States; Desai Sethi Urology Institute, Miami, FL 33136, United States; Department of Urology, Indiana University School of Medicine, Indianapolis, IN 46202, United States; Department of Urology, Indiana University School of Medicine, Indianapolis, IN 46202, United States; Desai Sethi Urology Institute, Miami, FL 33136, United States; Department of Urology, Baylor College of Medicine, Houston, TX 77030, United States; Department of Urology, Baylor College of Medicine, Houston, TX 77030, United States; Department of Urology, University of California, San Diego, San Diego, CA 92121, United States; Desai Sethi Urology Institute, Miami, FL 33136, United States; Department of Urology, Baylor College of Medicine, Houston, TX 77030, United States; Desai Sethi Urology Institute, Miami, FL 33136, United States; Department of Urology, University of Washington, Seattle, WA 98195, United States; Department of Urology, Indiana University School of Medicine, Indianapolis, IN 46202, United States; Department of Urology, Baylor College of Medicine, Houston, TX 77030, United States; Desai Sethi Urology Institute, Miami, FL 33136, United States; Desai Sethi Urology Institute, Miami, FL 33136, United States; Department of Urology, University of California, Irvine, Orange, CA 92868, United States

**Keywords:** Peyronie’s disease, collagenase *Clostridium histolyticum*, sexual and gender minorities, men who have sex with men

## Abstract

**Background:**

While prior studies have demonstrated that men who have sex with men (MSM) may disproportionately experience distress due to Peyronie’s disease (PD), limited evidence exists regarding the outcomes of these men when treated with collagenase *Clostridium histolyticum* (CCH) injections.

**Aim:**

To investigate the outcomes of MSM undergoing CCH injections for PD.

**Methods:**

A retrospective analysis was performed for 790 patients undergoing CCH injections for PD at 5 academic centers within the United States, including data pertaining to demographic information, sexual orientation, penile curvature characteristics, treatment characteristics, and treatment-related outcomes.

**Outcomes:**

The impact of sexual orientation on treatment-related satisfaction, efficacy, and complication rates.

**Results:**

Of the 399 patients meeting the final inclusion criteria, 6.51% (*N* = 26) self-identified as MSM. The median absolute reduction in penile curvature was 15 degrees (interquartile range: 5-20 degrees), with 70.0% (*n* = 206) reporting feeling satisfied with their treatment. Men who have sex with men patients were noted to have higher rates of corporal fracture following treatment (11.5% vs. 1.36%, *P* = .011), though sexual orientation was not associated with statistically significant changes in treatment satisfaction, penile curvature reduction, or rates of complications requiring operative intervention.

**Clinical Implications:**

An improved understanding of the outcomes of MSM receiving CCH injections may improve counseling regarding and management of PD for a vulnerable population of patients.

**Strengths and Limitations:**

This study offers some of the first data examining outcomes for PD in MSM patients. However, the retrospective and multicohort nature of the study, as well as its small cohort size, may result in impactful bias.

**Conclusion:**

In our trial, MSM patients receiving CCH were with similar treatment efficacy, treatment satisfaction, and rates of operative complications, suggesting that CCH is an efficacious and safe treatment for MSM patients.

## Introduction

Peyronie’s disease (PD) is characterized as the formation of fibrotic plaque or scar over the tunica albuginea, often associated with penile curvature, loss of penile length, and changes in penile girth.^1^^,^^2^ Within the United States, it is estimated that 0.8% of men over the age of 18 years have been treated for, or received a formal diagnosis of PD.^2^ Recent trials have demonstrated that a larger percentage, up to 6%, of men may present with some degree of penile curvature.^3^ Those with PD often present with a constellation of symptoms including penile curvature, penile deformity, pain, erectile dysfunction and difficulties with penetrative intercourse.^1^ The symptoms of PD may additionally cause significant distress on patients, with surveys linking PD with depression, negative body image, relationship impairments, and a loss of sexual confidence.^4^^,^^5^

Of those with PD, men who have sex with men (MSM) may be at a disproportionately high risk of psychosocial dysfunction due to PD. Men who have sex with men, who make up to 5.2% of the population of adult men in the United States, have previously been shown to experience higher rates of sexual dysfunction at baseline.^6^ Regarding those with PD specifically, MSM have previously been shown to present for treatment earlier than men who do not have sex with men (MNSM), possibly indicative of MSM experiencing a higher degree of bother from PD symptoms.^7^ Likewise, survey studies have demonstrated that MSM with PD noted worse scores on the sexual relationship subdomain of the SEAR questionnaire, noted lower confidence with erectile function and sexual performance, scored worse on patient and partner satisfaction scales, demonstrated worse self-esteem, and were less likely to initiate intercourse.^8^

Intralesional collagenase *Clostridium histolyticum* (CCH) offers patients a nonoperative treatment for this distressing condition. Collagenase *Clostridium histolyticum* is a mixture of 2 collagenases delivered by injection to the penile plaque in PD and is FDA approved for the treatment of penile curvature in PD.^9^ Collagenase *Clostridium histolyticum* has been associated with significant improvements in penile curvature compared to placebo, with improvements of 17-18 degrees, or a curvature reduction of ~34%, when administered in a series of 4 cycles of paired injections with manual modeling.^9^^,^^10^ While the use of CCH has been associated with high rates of patient satisfaction previously,^11–16^ little is known regarding the treatment satisfaction rates for MSM. By conducting a review of outcomes from a large retrospective cohort of patients treated with CCH injections, the authors sought to answer: do MSM patients, recognized as being at heightened risk for psychosocial impairment in the context of PD, demonstrate treatment satisfaction comparable to the broader PD cohort?

## Materials and methods

A retrospective analysis was performed for 790 patients diagnosed with PD undergoing treatment with CCH injections between December 2013 through July 2025 at 1 of 5 academic centers within the United States. Men older than 18 years were included if diagnosed with PD using an in-office duplex ultrasound following the creation of a pharmacologically induced erection and undergoing treatment with CCH injections. Demographic information including age, patient-reported race, patient-reported sexual orientation, comorbidities, and past medical and urologic history were recorded. Treatment characteristics, including number of injections, use of manual modeling and/or device traction, treatment complications, and patient-reported treatment satisfaction were recorded. Treatment complications were noted as operative if requiring surgical intervention and were recorded based on provider documentation during follow-up visits. Recorded curvature characteristics included pretreatment and posttreatment penile curvature as measured by a urologist, patient-reported pretreatment curvature, curvature laterality, and the presence of hourglass deformity, tapering, multiplanar curvature, and plaque calcification. Patients were excluded from select analyses when sexual-orientation or the examined treatment outcomes were not reported. The impact of sexual orientation on treatment outcomes, including change in penile curvature, complication rate, and patient-reported treatment satisfaction rate, as recorded from provider documentation during follow-up visits, was assessed using simple linear and logistic regression, chi-squared test, Fisher’s exact test, and Wilcoxon-Mann–Whitney test for univariate analyses. All statistical analysis were performed using the Stata 19 software. Statistical significance was defined as a *P*-value of <.05. The study was performed in line with the Declaration of Helsinki and each site received approval from a local institutional review board with approved waivers for informed consent applied given the study’s retrospective cohort design.

## Results

Of the 790 patients screened, 399 (50.5%) met the inclusion criteria, with 383 (48.5%) being excluded for missing data on sexual orientation and an additional 8 (1.0%) being excluded for incomplete data relating to treatment protocol. The median age of patients included was 58 years (interquartile range [IQR]: 52-62). The median follow-up period following the last CCH injection was 8.4 months (IQR: 4.1-16.9 months). A total of 6.51% of patients (*N* = 26) self-identified as MSM. The demographic information of patients is summarized in Table 1. The median pretreatment curvature was 40 degrees (IQR: 30-45 degrees) and 45 degrees (IQR: 35-55 degrees) as reported by patients and measured by practitioners, respectively. Patients were found to have penile curvature involving the dorsal, ventral, left, and right penile shaft in 61.6% (*N* = 246), 9.02% (*N* = 36), 30.1% (*N* = 120), and 12.0% (*N* = 48) of patients, respectively. Patients received a median of 8 injections (IQR: 5-8 injections) from 1 of 12 practitioners, with 61.9% (*N* = 247) completing 4 cycles of paired injections. Overall, 76.4% (*N* = 305) engaged in home modeling following injections and 62.2% (*N* = 248) endorsed use of a traction device. The median posttreatment curvature was 30 degrees (IQR: 20-40 degrees) with a median absolute reduction in penile curvature of 15 degrees (IQR: 5-20 degrees), or an average curvature reduction of 29.8%. For those who were followed for posttreatment satisfaction (*N* = 303), 70.0% (*N* = 206) reported overall satisfaction with treatment. The rate of treatment-related adverse events is described in Table 2.

**Table 1:** Demographics of the study sample.

Characteristic	Percentage of patients responding (*N*)
Academic site	
One	41.6 (*n* = 166)
Two	4.5 (*n* = 18)
Three	11.0 (*n* = 44)
Four	24.3 (*n* = 97)
Five	18.5 (*n* = 74)
Race/ethnicity	
White	78.9 (*n* = 315)
Hispanic	9.3 (*n* = 37)
Black	4.8 (*n* = 19)
Asian	2.5 (*n* = 10)
Other	3.5 (*n* = 14)
Prefer not to respond	1.0 (*n* = 4)
Age	
20-29	0.5 (*n* = 2)
30-39	5.5 (*n* = 22)
40-48	12.0 (*n* = 48)
50-59	42.6 (*n* = 170)
60-69	33.6 (*n* = 134)
>70	5.8 (*n* = 23)
Comorbidities	
Diabetes	18.3 (*n* = 73)
Cardiovascular disease	3.8 (*n* = 15)
Kidney disease	3.5 (*n* = 14)
Chronic Obstructive Pulmonary Disease	6.0 (*n* = 24)
Heart failure	1.3 (*n* = 5)
Peripheral vascular disease	2.3 (*n* = 9)
Cerebrovascular event	2.0 (*n* = 8)
Solid malignancy	13.8 (*n* = 55)
Liver disease	3.3 (*n* = 13)
HIV/AIDS	1.8 (*n* = 7)
Hematologic malignancy	1.0 (*n* = 4)
Without comorbidities	57.9 (*n* = 231)

**Table 2:** Summary of treatment-related adverse events.

Adverse event	Percentage for MSM (*N*)	Percentage for MNSM (*N* = 373)
Bruising	19.2 (*n* = 5)	29.2 (*n* = 109)
Swelling	15.4 (*n* = 4)	24.7 (*n* = 92)
Pain requiring medication	3.9 (*n* = 1)	8.6 (*n* = 32)
Infection	0.0 (*n* = 0)	0.8 (*n* = 3)
Hematoma	3.9 (*n* = 1)	8.0 (*n* = 30)
Hematoma requiring drainage	0.0 (*n* = 0)	1.1 (*n* = 4)
Penile fracture^*^	11.5 (*n* = 3)^*^	1.3 (*n* = 5)^*^
Operative penile fracture	7.7 (*n* = 2)	0.3 (*n* = 1)
Penile length loss	3.9 (*n* = 1)	1.9 (*n* = 7)
New erectile dysfunction	3.9 (*n* = 1)	2.9 (*n* = 11)

Abbreviations: MSM, men who have sex with men; MNSM, men who do not have sex with men. ^*^ = *P* < .05.

On univariate analysis, MSM were more likely to have a history of HIV/AIDS (26.9% vs. 0%, *P* < .001), presented closer to symptom onset (9 months vs. 13 months, *P* < .001), used intracorporal injections for erectile dysfunction (19.2% vs. 5.71%, *P* = .005), and experienced a corporal fracture following treatment (11.5% vs. 1.36%, *P* = .011). Men who have sex with men were less likely to have a history of solid tumors (0% vs. 17.4%, *P* = .035) and used traction devices between injections (38.5% vs. 63.8%, *P* = .010). A summary of variables as differentiated by sexual orientation on univariate analysis is provided in Table 3.

**Table 3:** Comparison of patient characteristics and outcomes by sexual orientation on univariate analysis.

Variable	MSM (*N* = 26)	MNSM (*N* = 373)
Prior treatment for penile curvature Active phase Median delay from symptom onset to treatment^*^ Multiplanar penile curvature Hourglass deformity Median number of injections Treatment completion rate Average change in penile curvature Treatment satisfaction rate	37.3% (*n* = 139) 0.0% (*n* = 0) 9 months^*^ 23.1% (*n* = 6) 16.0% (*n* = 4) 8 61.5% (*n* = 16) 14.1±13.1 degrees 82.6% (*n* = 19)	19.2% (*n* = 5) 6.0% (*n* = 22) 13 months^*^ 24.7% (*n* = 88) 20.9% (*n* = 72) 8 62.0% (*n* = 222) 14.8±16.1 degrees 66.8% (*n* = 187)

Abbreviations: MSM, men who have sex with men; MNSM, men who do not have sex with men. ^*^P < .05.

On univariate analysis, subjective posttreatment satisfaction was only significantly associated with changes in provider (*P* = .036) and with change in penile curvature (odds ratio = 0.93, *P* < .001). However, no associations were noted between treatment satisfaction and sexual orientation (*P* > .05). Change in curvature following treatment was significantly associated with provider (*P* < .001), race (*P* = .005), age (β = −0.27, *P* = .006), and pretreatment curvature (β = −0.43, *P* < .001). Change in penile curvature was not significantly associated with either number of injections received nor sexual orientation (*P* > .05).

## Discussion

In our retrospective cohort analysis, MSM demonstrated post-treatment satisfaction rates and absolute changes in penile curvature comparable to those of MNSM. The average absolute change in penile curvature for MSM of 14.1 degrees, or average curvature reduction of 29.3%, matches that reported in the prior literature. In the IMPRESS I and II trials, over 400 men underwent treatment with 8 injections of CCH with a completion rate of 78.8%, higher than that of the currently reported cohort. Following treatment, patients were recorded to have similar degrees of curvature improvement of 17±14.8 degrees of absolute curvature reduction, or a 34% reduction in penile curvature.^9^ Likewise, in the large, open label study conducted by Levine et al., 348 men underwent a series of 4 injection cycles with a 77.8% completion rate and noted an average reduction in penile curvature of 18.3±14.0 degrees, or a 34.4% reduction in penile curvature.^10^ Similarly, in their single arm prospective trial of 135 patients receiving CCH for PD, Cocci et al. reported a median change in penile curvature of 20 degrees, or a 44% curvature reduction.^14^ While the above trials reported large percentage changes in penile curvature with CCH, other trials report more modest improvements. In their retrospective cohort study of 114 patients undergoing CCH for PD with a 44% completion rate, Flores et al. recorded a smaller average curvature improvement of 7 degrees.^17^ Similarly, in their prospective registry of 228 patients receiving CCH with or without traction devices, Alom et al. reported an overall composite change of 18.6 degrees, which resulted in only a 27.4% change in penile curvature.^15^ Matching closely the degree of curvature improvement to our data are the data from Hellstrom et al., in which 918 men receiving CCH were retrospectively reviewed and found to have a posttreatment change in penile curvature of 14.5±14 degrees.^18^ As such, our data present absolute and percentage reductions in penile curvature which fall within the range of improvement previously reported in the literature. While the improvement in penile curvature with CCH did not match the high levels of improvement as described in the IMPRESS I and II trials or as reported by Levine et al., it is notable that these trials demonstrated higher treatment completion rates at 78.8% and 77.8%, respectively, compared with 61.9% in our cohort. While in our cohort the number of injections received was associated with treatment satisfaction, this variable was not found to be associated with change in penile curvature. However, the number of cycles received was previously identified to be an independent predictor of change in penile curvature when examining larger cohorts.^18^ Given this previously identified association, it may be that the lower completion rate in our cohort could explain the differences in penile curvature change when comparing our cohort to prior studies. The discrepancy between our findings and those of Hellstrom et al. regarding the impact of the number of injections received and change in penile curvature may be due to the use of injections as opposed to cycles as an independent variable, or due to the smaller cohort size studied.

In our study, 82.6% of MSM and 66.8% of MNSM noted posttreatment satisfaction, though this difference was not found to be statistically significant. These high rates of posttreatment satisfaction with CCH match, if not exceed, the subjective reports of patients undergoing CCH in prior studies. In a survey study conducted by Kaminetsky et al., 45 patients following a phase 2b trial on CCH for PD were found to have a 51.1% satisfaction rate following treatment, with 48.9% defining the treatment as overall successful.^11^ In a similar survey conducted by Ziegelmann et al., 41 of 52 patients (79%) undergoing CCH found their treatment to be clinically meaningful.^12^ Measuring a similar metric, Alom et al. and Alom et al. reported between a 79% and 93% rate and 86.6% rate of reaching a subjectively meaningful response following treatment with CCH.^13^^,^^15^ Measuring satisfaction scores, Cocci et al. reported a median satisfaction score of 8/10 for their 135 patients receiving CCH.^14^ As with Ziegelmann et al., subjective improvement following treatment was associated with absolute curve improvement.^12^

Regarding complications, 30.8% of MSM and 35.9% of MNSM had an adverse event, including swelling, bruising, hematoma, infection, loss of penile length, erectile dysfunction, pain requiring prescription medication, and penile fracture, though the difference in complication rates were not significant. While the rate of penile fractures in MSM exceeded that of MNSM to a statistically significant degree, the rate of operative repairs for penile fracture did not differ between groups. Of note, the overall complication rate was far less than those reported in the IMPRESS trials and Levine et al., which reported complication rates between 84.2% and 92%.^9^^,^^10^ However, these differences are likely due to the prospective nature of the latter studies and limitations secondary to the retrospective chart review nature of the current study. Examining hematomas and penile fractures, 2 adverse events that are often highlighted in studies to a larger degree, the overall cohort in our study was with 8 penile fractures (2.01%) and 31 hematomas (7.77%), for which 3 (0.75%) and 4 (1.00%) patients were managed surgically, respectively. In the IMPRESS trials, a smaller number of penile fractures and hematomas were recorded at 0.54% each, though all penile fractures were managed surgically.^9^ Similarly small rates of hematoma at 2% and penile fracture at 0.29% were noted in Levine et al.^10^ While Hellstrom et al. reported higher hematoma rates at 5.1%, the rates of penile fracture in their respective cohort study similarly were smaller than our reported data at 0.8%.^18^ Despite these results, other studies have reported more frequent rates of these complications. In Alom et al., up to 4% of patients undergoing CCH with concurrent traction device use experienced a penile fracture.^13^ Likewise, in their small cohort of 49 men receiving CCH for PD, Yang et al. reported a hematoma rate of 8.16% and fracture rate of 2.04%, more closely matching our reported outcomes.^16^ Of note, the current study is the first to demonstrate that a specific demographic group may be at a higher risk of penile fracture with CCH. Notably as well, this is the first study to present an association between complications and improved subjective satisfaction, despite no association being noted between complications and improved change in penile curvature. As these associations have not previously been described, future prospective studies would be required to reproduce this effect prior to speculation of any causality behind these effects or prior to the incorporation of these results into pretreatment patient counseling. Should the association between an increased rate of penile fractures and sexual orientation be confirmed, one possible explanation could be that MSM are engaging more often in penetrative anal intercourse, which is theorized to be associated with higher axial forces on the penile shaft compared with vaginal penetration.^19^ However, as prior studies demonstrate that MSM are more likely to engage in partnered masturbation and oral intercourse over anal intercourse,^20^ and as not all MSM engaging in anal intercourse are the penetrative partner, this may not be a complete explanation. As MSM are thought to be at higher risk of psychosocial impacts from PD, it may also be the case that MSM engage in more frequent or more aggressive manual modeling and device traction use, which in turn could explain the higher rate of penile fractures. However, in our own data, the rate of manual modeling was not statistically different between MSM and MNSM, and MSM were statistically less likely to use traction devices than MNSM. As such, this explanation appears to be invalidated by our cohort’s data.

Numerous limitations exist for the current study. The study’s design as a multicenter, retrospective study opens the study to the risk of various bias. As data was obtained from a retrospective chart review, data accuracy was dependent on the way in which practitioners recorded the included variables while charting, which may have also differed between providers. One such way in which the data may be affected in this regard is in the measurement of treatment satisfaction. As satisfaction was measured subjectively and as recorded in office visit notes, as opposed to with an objective scale, variations in how each practitioner recorded patient satisfaction may have impacted outcomes. Likewise, variations in the way in which each practitioner administered injections, counseled patients on home modeling and/or traction, and measured penile curvature could impact the findings of the study. The risk of bias due to these factors is notably high given that on analysis, the provider seen was found to have a statistically significant impact on posttreatment satisfaction. The large dropout percentage from the initial cohort (49.5%), due to incomplete documentation of sexual orientation for many patients, is an additional limitation of the study. Much of this dropout is likely due to providers inconsistently inquiring on a patient’s sexual orientation and sexual practices, a phenomenon which is previously well-documented in the literature.^21^ As a result of this dropout, and the resulting small cohort of MSM patients, a multivariable analysis could not be performed. As such, the data may be at risk of bias or confounding effects. Given the retrospective and deidentified nature of the study, further inquiries into patterns of measuring sexual orientation at each center over time were not possible. Likewise, the lack of information on sexual practices makes an analysis on the association between penile fractures and specific sexual activities impossible in the current study. In the future, the use of a large-cohort, prospective study, for which unified treatment and curvature measurement protocols are enacted, and for which satisfaction is measured through objective scales, could address the above limitations.

## Conclusion

Peyronie’s disease can be a debilitating condition, not only causing penile curvature and impairments with sexual function, but also placing patients at risk of relationship impairments and depressed mood. While MSM may be at a disproportionately high risk of experiencing the negative psychosocial impacts of PD, little is known regarding this group’s outcomes with PD treatment. Using a retrospective cohort from 5 academic centers, the outcomes of MSM with PD undergoing treatment with CCH were recorded. Overall, MSM receiving CCH were with similar changes in penile curvature and satisfaction rates to MNSM. While MSM experienced higher rates of corporal fractures than MNSM, rates of operative penile fractures remained low in both groups. As such, CCH appears to be well tolerated and associated with high treatment satisfaction in MSM.
